# Neuroprotective Strategies for Traumatic Brain Injury: Improving Clinical Translation

**DOI:** 10.3390/ijms15011216

**Published:** 2014-01-17

**Authors:** Shruti V. Kabadi, Alan I. Faden

**Affiliations:** Department of Anesthesiology, Center for Shock, Trauma and Anesthesiology Research (STAR), National Study Center for Trauma and EMS, University of Maryland School of Medicine, Baltimore, MD 21201, USA; E-Mail: skabadi@anes.umm.edu

**Keywords:** experimental head injury, clinical trial design, translational challenges, multipotential neuroprotective approaches, programmed cell death, caspase-dependent and AIF-mediated cell death, microglial and astrocyte activation, autophagy

## Abstract

Traumatic brain injury (TBI) induces secondary biochemical changes that contribute to delayed neuroinflammation, neuronal cell death, and neurological dysfunction. Attenuating such secondary injury has provided the conceptual basis for neuroprotective treatments. Despite strong experimental data, more than 30 clinical trials of neuroprotection in TBI patients have failed. In part, these failures likely reflect methodological differences between the clinical and animal studies, as well as inadequate pre-clinical evaluation and/or trial design problems. However, recent changes in experimental approach and advances in clinical trial methodology have raised the potential for successful clinical translation. Here we critically analyze the current limitations and translational opportunities for developing successful neuroprotective therapies for TBI.

## 1. Introduction

Traumatic brain injury (TBI) is a major cause of death and disability in humans. The incidence of TBI in the United States is at least 1.7 million annually with an estimated 5 million patients experiencing long-term complications (Centers for disease control and prevention (CDC), facts about traumatic brain injury). Use of improvised explosive devices in war zones have resulted in increasing numbers of blast-related head injuries in both military personnel and civilians. Indeed, TBI has been considered a “signature injury” of the wars in Iraq and Afghanistan [1,2]. More recently, there has been increased recognition of the frequency and consequences of concussive brain injury in athletes and military personnel [3–5]. According to the CDC, the annual estimated direct and indirect medical costs of TBI are close to $76.5 billion in the United States. But TBI is a global problem [6].

In order to better understand the pathobiology of TBI and evaluate potential therapeutic approaches, various animal models have been developed and characterized. Each is intended to mimic certain components of clinical TBI, recognizing that it is difficult to establish consistent models that include most or all of the factors that contribute to post-traumatic tissue damage. Importantly, TBI represents perhaps the most heterogeneous of neurological disorders; in addition to severity, differences across patients may reflect location, invasive *versus* non-invasive insults, focal *versus* diffuse, presence or absence of intracranial bleeding, as well as differences in gender, genetic predisposition, and presence or absence of certain co-morbidities. Thus, although the animal models of TBI have generated valuable information on delayed biochemical changes that lead to behavioral dysfunction and provided the experimental basis for treatment strategies, clinical trials of drugs showing preclinical improvements have uniformly failed, reflecting in part the major methodological differences between preclinical and clinical modeling and evaluation [7–10]. Potential caveats about animal modeling include questions about how well they simulate clinical pathophysiology, especially diffuse axonal injury; use of anesthetics resulting in potential drug-drug interaction issues; failure in most cases to demonstrate that proposed preclinical mechanisms reflect those in humans, use of genetically identical subjects and failure to address gender, injury severity, species, strain or age-related differences in most pre-clinical evaluations; and choice of outcomes that differ from those used clinically.

Another major methodological issue has been the historical focus on using treatments directed toward single injury mechanisms, although clearly secondary injury is multi-factorial. More recently, the focus has shifted to address the need to modify multiple targets, either through combination therapies or through use of single agents that modulate multiple key secondary events.

## 2. TBI: A Complex and Chronic Disorder

Both human and animal studies have indicated that TBI leads to chronic biochemical events that play a significant role in exacerbating head injury-induced tissue loss and neurological deficits. Head injury causes cell death and neurological dysfunction first by both direct physical tissue disruption (primary injury), as well as from delayed and potentially reversible molecular and cellular pathophysiological mechanisms that cause progressive white and grey matter damage (secondary injury) [11,12]. Such delayed injury begins within seconds to minutes after trauma, may continue for weeks or months or potentially years [12], and eventually may be responsible for a significant component of the chronic neurodegeneration and neurological impairment following TBI [10].

The primary injury can be described as the mechanical damage occurring at the time of trauma to the neurons, axons, glia and blood vessels through shearing, tearing and stretching [13,14]. Such events pave the way for secondary pathophysiological cascades that include biochemical, metabolic and physiological changes such as spreading depression, ionic imbalance, release of excitatory neurotransmitters, mitochondrial dysfunction, and activation of inflammatory and immune processes [8–10,15], among others. Some of the more important secondary injury mechanisms involve activation of neuronal cell death pathways, microglial and astrocyte activation, and neurotoxicity. Notably, chronic inflammation following CNS trauma has provided a mechanistic link between acute and chronic neurodegeneration [16]. Preclinical studies have indicated that sustained microglial and astrocyte activation after CNS trauma may play a role in the chronic neurodegeneration and loss of neurological function [17,18]. Although both neuroprotective and neurotoxic microglial phenotypes have been described [19–21], microglial activation and the release of associated inflammatory factors has been proposed as an important contributing factor in chronic neurodegenerative disorders, including Alzheimer’s Disease [22]. Furthermore, previous studies have indicated that sustained microglial activation after CNS trauma may play a role in neuronal cell loss following the release of neurotoxic molecules such as NO [17,23]. Therefore, TBI must not be considered an acute or static disorder, but a complex and chronic neurodegenerative condition. Interestingly, the delayed nature of such injury has suggested the existence of a substantially longer therapeutic window for intervention after TBI, which challenges the traditionally-accepted view that TBI-induced damage can only be reversed within a few hours of trauma. Despite considerable success in elucidating secondary injury mechanisms, more than 30 phase III prospective trials of targeted drug therapies that showed promise in experimental models, have failed to generate favorable results under clinical settings [24–26]. Therefore, it is important to critically review potential factors contributing to such failed clinical translation.

## 3. Translational Challenges and Strengthening Preclinical Support

Potential factors contributing to failed translation may include the following (Table 1).

- Failure to appreciate the complexity and diversity of secondary injury mechanisms;
- Inadequate attention to such potential confounding factors as species, strains, gender and age in drug evaluation;
- Use of anesthetics in animal models;
- Inability of the drugs to adequately cross the blood-brain barrier and reach therapeutic concentrations in the brain;
- Failure to assess clinically relevant therapeutic windows or clinically-relevant behavioral outcomes;
- Pharmacogenetic/epigenetic variability of heterogeneous patient populations;
- Lack of predictive biomarkers;
- Inadequate sample sizes and/or failure to utilize the most effective experimental design to increase power.

Many animal models of experimental TBI have been developed and characterized using different types of mechanical forces. Experimental models of brain injury use mechanical force to cause static or dynamic brain trauma [27,28]. The static models rely on amplitude and duration of mechanical force to cause trauma-induced morphological and functional impairments [27–29]. Dynamic brain injury can be induced via a defined amplitude, duration, velocity and/or acceleration of the mechanical force [27]. However, with a few exceptions, all animal models of trauma require anesthetizing the animals for inducing head injury. Anesthetic agents have been demonstrated to exhibit neuroprotective or neurotoxic effects that can interact/interfere with pharmacological actions of the drug under investigation [30,31]. The clinical relevance of *in vivo* models has been questioned because the same model can induce markedly variable injury and outcomes across different strains [32], which can be particularly important in studies using transgenic animals, because of the impact of the type of backcrossing. Most animal models use rodents because of cost and animal rights issues. Yet rodents have substantially smaller brains with less white matter than humans, which may result in less diffuse axonal injury [33]. Problems using larger gyrencephalic animals include not only cost but also limitations of established behavioral outcomes or antibodies to address mechanism (e.g., in sheep). Nonetheless, it is desirable to demonstrate neuroprotection in larger gyrencephalic species before moving to clinical trials.

The effects of gender on injury mechanisms remain controversial. Considering the putative neuroprotective effects of estrogen and progesterone, it has been suggested that females are somewhat protected from secondary injury compared to males. However, recently, several clinical TBI studies have reported worse outcomes in females *versus* males following mild TBI or sports concussion injury [34–38]. A study following sports-related concussion suggested that female athletes experienced a greater decline in reaction time and significantly higher incidence of post-concussion symptoms related to emotional, physical or sleep domains, and cognitive impairment than males [38]. Covassin and Bay [37] reported a correlation between significant worsening in verbal memory and motor processing speed scores, as well as elevated chronic stress levels in female patients after mild to moderate TBI. Gender may also be an important factor with respect to treatment of TBI. Early intervention with enriched environment has been observed to exhibit a beneficial effect on cognitive recovery in male but not in female rats following experimental brain injury [39]. Similarly, methylphenidate treatment following TBI using the controlled cortical impact (CCI) model significantly improved spatial memory acquisition and retention in the Morris water maze test in injured male *versus* female rats [40]. Therefore, gender-specific differences may have important implications for understanding the pathophysiology and treatment of TBI.

Experimental design and statistical analyses of preclinical studies differs significantly from data management and analysis under clinical trials. One difference may be the *post hoc* deletion of animal subjects because of an outlier in outcomes or animal subjects not meeting the injury criteria; in contrast clinical trials use intent-to-treat paradigms, where data are not excluded because of errors in drug preparation or administration, and/or variability in the degree of injury severity [9,10]. Inadequate sample sizes have been an important problem with many prior clinical TBI trials, in which expected differences have been set as high as 20% or more. Power analyses have often assumed very large and unrealistic treatment effects. In contrast, the more recent “Clinical Randomization of an Antifibrinolytic in Significant Hemorrhage/Head Injury” (CRASH) trials evaluated 10,000 patients, so as to potentially identify even a 1% treatment effect [41,42].

The selection of appropriate therapeutic window of drug administration is critical in animal studies, but often lacks clinical relevance. In most animal studies, treatment is administered either before or within a few minutes to an hour after trauma. It is impractical to replicate the same early administration paradigm in clinical cases because of evaluation and informed consent issues. Generally, preclinical studies evaluating the efficacy of pharmacological agents for TBI do not assess the pharmacokinetics or pharmacodynamics related to the drugs administered, and thus do not attempt to optimize or identify effective brain concentrations required. In addition, the specificity of the treatment target is often questionable because the pharmacological agents have secondary treatment effects and may modulate other molecular pathways. Strategies such as use of structurally different modulators with similar effects or simultaneous studies with knockout models may resolve these issues. However, such complementary studies are often not performed because of the time and cost involved.

There are other suggestions for strengthening preclinical support for potential novel neuroprotective strategies. Demonstrated effects should be robust, not only significant, and should be demonstrated across multiple experimental models and species. Studies should be replicated across laboratories. Behavioral, as well as histological or imaging outcomes should be demonstrated. Dose-response, brain penetration, pharmacokinetic and pharmacodynamic studies should be performed in animals, with optimal dosing and dose schedules established. Finally, the therapeutic window for any proposed treatment should be at least 6–8 h.

## 4. Advances in Clinical Trials Design

The current functional classification used in clinical trials of TBI involves a 15-point Glasgow Coma Scale (GCS) [10]. TBI patients are classified as suffering from mild (14–15), moderate (9–13) or severe (3–8) head injury. Because of the arbitrary nature of the GCS classification, more recently the extended Glasgow Outcome Score (GOS-E) with additional endpoints of assessment has been proposed and accepted as the clinical measure in TBI patients [10]. Interestingly, the importance of using expanded categories has often been negated by *post hoc* categorization into “good” and “bad” outcomes. A more comprehensive and symptom-based classification is required, which includes evaluation of specific behavioral outcomes such as cognitive and motor functions, quality of life, and physiological- and imaging-based biomarkers [14].

The methodology and design of clinical trials has been significantly revised to increase patient recruitment rates by expanding the inclusion criteria after carefully considering the specific mechanisms of action of the treatment under investigation [43]. The International Mission on Prognosis and Clinical Trial Design in TBI (IMPACT) group has provided such recommendations to improve clinical trial design and analysis [43]. Recommendations include incorporation of pre-specified covariate adjustments to mitigate the effects of heterogeneity in TBI populations and use of an ordinal statistical approach, using either sliding dichotomy or proportional odds methodology [43]. In addition, the recommendations include incorporation of pre-specified covariate adjustments to mitigate the effects of heterogeneity and use of an ordinal approach, based on either sliding dichotomy or proportional odds methodology while performing statistical analysis [43]. More recently, an adaptive design methodology in clinical research and developments was introduced and is being followed to allow more flexibility and better efficiency. An adaptive design, also known as a flexible design can be defined as a plan that permits adaptations or modifications to the methodology and/or statistical procedures of a clinical trial after its initiation without compromising the validity and integrity of the trial [44–46]. Furthermore, efforts have begun internationally to markedly expand observational datasets from TBI patients in order to identify common data elements (CDE), to develop tools for defining sub-groups, and to identify surrogate biomarkers that can facilitate future clinical investigation (www.nindscommondataelements.org/TBI.aspx) [10,24].

## 5. Selective *versus* Multipotential Neuroprotective Strategies

Most of the failed clinical trials used treatments targeted single proposed injury mechanisms such as excitotoxicity mediated by ionotropic glutamate receptors. Yet secondary injury reflects a cascade of often interactive factors/mechanisms. Clinical trials in cancer and infectious diseases have repeatedly shown that multidrug treatments may be required to produce optimal therapeutic results. However, combination drug therapies in TBI would be very expensive, and experimental studies have demonstrated that combinations of highly effective treatments may show poorer outcomes that optimal treatment with single agents, potentially reflecting unanticipated drug-drug interactions [47]. Therefore, the current experimental research focus is on development of single treatment strategies that have multipotential effects on various secondary injury mechanisms (Table 2).

Naturally occurring hormones such as corticosteroids, thyrotropin-releasing hormone and progesterone were some of the first agents to be evaluated for their multipotential pharmacological effects in TBI models. Progesterone has been shown to exhibit neuroprotective effects in animal models of SCI [48], stroke [49] and TBI [50]. It attenuates glutamate excitotoxicity [51], membrane lipid peroxidation [52], apoptotic pathways [53], and diffuse axonal injury [54]. Two randomized, double-blinded, placebo-controlled phase II clinical trials for progesterone have been conducted that showed trends towards improvement in outcomes by progesterone treatment [55,56]. However, experimental TBI studies have resulted in mixed results [57] and a systematic review noted that many of the experimental studies were of poor methodological quality, therapeutic window studies were narrow, and there was statistical evidence of bias in the experimental TBI as opposed to experimental stroke work in the field [58]. There have been two phase III multi-center clinical trials. The ProTECT phase III trial (NINDS/NIH) used initiation of progesterone via intra-venous (i.v.) administration within 4 h of TBI, continuing for 72 h in patients with moderate to severe TBI [59,60]. The SyNAPSE phase III trial (BHR Pharma) involves administration of i.v. infusion of BHR-100 (progesterone) initiated within 8 h in severe TBI patients [59,60]. The ProTECT trial was recently terminated, because of lack of data supporting effectiveness. The other clinical trial continues.

Thyrotropin-releasing hormone also has multiple neuroprotective effects including increased cerebral blood flow and metabolism; and attenuating levels of peptidyl leukotrienes, platelet-activation factor, endogenous opioids and glutamate [61]. Although numerous animal TBI studies have demonstrated neuroprotective potential of thyrotropin-releasing hormone, no large randomized clinical trial has yet been conducted.

Amongst naturally-based hormones with neuroprotective effects in TBI models, erythropoietin—a glycoprotein hormone and a cytokine that controls erythropoiesis, has shown promise in preclinical as well as clinical settings. Erythropoietin has been shown to exhibit anti-excitotoxic, anti-oxidant, anti-edematous, and anti-inflammatory effects in TBI models [62,63]. In addition to exhibiting beneficial neuropharmacological effects, erythropoietin shows a favorable clinical pharmacokinetic profile with high CNS penetration and bioavailability [62]. However, it may cause thromboembolic effects because of its erythropoiesis-inducing properties. To overcome these adverse biological effects, a structural derivative of erythropoietin (carbymylated erythropoietin) and a peptide based on the structure of the Helix B segment of erythropoietin (pyroglutamate Helix B surface peptide), have been developed; they do not bind to the classical erythropoietin receptor but exhibit similar neuroprotective effects without causing thromboembolic actions [62]. The first randomized clinical trial of erythropoietin in blunt trauma patients was conducted to determine the effects of the treatment on cell death and daily S100B and neuron specific enolase (NSE) levels [92]. However, erythropoietin treatment failed to have any impact on S100B or NSE levels. A randomized, placebo-controlled, double-blinded clinical trial of erythropoietin administration is underway; organized by the Australian and New Zealand Intensive Care Research Center in patients with moderate or severe TBI, it will evaluate long-term neurological outcomes.

Induction of hypothermia by lowering of the body temperature to between 32 and 35 degree Celsius (°C) provided neuroprotection in several animal TBI models [64–67]. Small variations in brain temperature can critically influence the extent of histopathological damage caused by an injury to the brain. Mild hypothermia protects the brain from ischemia and TBI, while mild hyperthermia worsens neurological outcomes [65,67,68]. Thus, selective brain cooling may be advantageous in attenuating the detrimental consequences of brain injury [64,65,67,68]. Furthermore, post-traumatic hypothermia caused a significant reduction in the number of necrotic cortical neurons and the contusion volume in rats after experimental TBI [66]. In clinical studies hypothermia has been shown to reduce intra-cranial pressure [69–71], cerebral metabolic rate [72], and increase brain and jugular vein oxygenation [71]. However, clinical trials have shown conflicting results and a randomized multi-center study reported no significant differences in neurological recovery or deaths in patients subjected to hypothermia when compared to normothermia [73]. Some investigators have argued that better results may occur under different experimental conditions- such as use in selected sub-populations, earlier treatment and/or for longer duration or to different temperatures, and altered rewarming protocols. Others have suggested that combining hypothermia with pharmacological neuroprotection may serve to increase drug effectiveness or therapeutic window. Because of absent convincing new clinical studies, hypothermia as a neuroprotective strategy for clinical TBI must be viewed cautiously.

### 5.1. Recent Advances in Neuroprotective Strategies for TBI

#### 5.1.1. Diketopiperazines

A class of novel cyclic dipeptides (diketopiperazines) has shown remarkable neuroprotective potential both *in vitro* and in rodent TBI models. Diketopiperazines are structurally similar to a physiologically-active metabolic product of thyrotropin-releasing hormone. One of the compounds, 35b, shows strong neuroprotective effects across TBI models, improving functional recovery and reducing lesion volume after fluid percussion injury (FPI) in rats [93] or controlled cortical impact in mice [94]. Three other diketopiperazines (compounds 144, 606 and 807) showed similar behavioral and histological protective effects after mouse CCI [95]. Another class of diketopiperazines, cyclo-l-glycyl-l-2-allylproline (NNZ 2591), improved functional recovery and histological outcomes, and attenuated apoptotic pathways and microglial activation in rats after hypoxic-ischemic brain injury [74]. 35b treatment reduced the expression of multiple cell cycle members, as well as calpain and cathepsin, while increasing expression of two potent endogenous neuroprotective factors-brain derived neurotrophic factor (BDNF) and heat shock protein (HSP) 70 [95]. These mutipotential drugs exhibit a clinically relevant therapeutic window of at least 8 h, show good brain penetration after systemic treatment and have a favorable safety profile- making them promising candidates for future clinical trials.

#### 5.1.2. SUR1-Regulated NC_Ca-ATP_ Channel Inhibitors

The up-regulation of sulfonylurea receptor 1 (SUR1)-regulated NC_Ca-ATP_ channels in microvascular endothelium has been implicated in models of CNS ischemia and trauma a secondary injury mechanisms [96]. Administration of the SUR1 antagonist glibenclamide reduced edema, secondary hemorrhage, inflammation, apoptosis and lesion size, and improved functional recovery after experimental TBI [75]. Given that glibenclamide is already used in humans as a hypoglycemic therapy, it has fast-track potential for clinical trials.

#### 5.1.3. Statins

Statins or 3-hydroxy-3methylglutaryl coenzyme A (HMGCoA) inhibitors attenuate cholesterol biosynthesis and have multipotential neuroprotective effects [97,98]. Statins have shown neuroprotection in TBI models. They limit production and expression of inflammatory mediators such as interleukin-6 (IL-6), tumor necrosis factor-alpha (TNF-α), and intracellular adhesion molecule 1 (ICAM-1); reduce glial cell activation and cerebral edema, and increase blood-brain barrier integrity [76,77]. These anti-inflammatory effects exhibited by the statins may in part be mediated by inhibition of toll-like receptor 4 and nuclear factor κB (NF κB) [76].

One of the primary advantages of statins is that these drugs have a wide therapeutic window, with treatment 24 h after TBI improving functional deficits and neuronal recovery [99,100]. In addition, statins are well tolerated and have been extensively used clinically [97,98]. A clinical trial with rosuvastatin in TBI patients showed improvement in amnesia and disorientation-related outcomes [101]. Phase II clinical trials for the administration of rosuvastatin and atorvastatin to TBI patients have been planned [10].

#### 5.1.4. Cyclosporin A

Cyclosporin A is an immunosuppressant drug that attenuates mitochondrial failure by binding to cyclophilin D and stabilizing mitochondrial permeability transition pore (mPTP) [102,103]. In addition to preserving mitochondrial function, cyclosporin A and its analogs inhibit lipid peroxidation and oxidative stress, particularly by attenuating pathways that involve mitochondrial proteins [78,79]. In TBI models cyclosporin A reduces axonal damage and decreases lesion size [102–105]. Like statins, cyclosporin A has a longer therapeutic window of 24 h [104] and is FDA-approved for other clinical purposes. A randomized, placebo-controlled, double-blind clinical trial of cyclosporin A in patients with severe TBI showed significantly reduced glutamate concentration and lactate/pyruvate ratios, and increased mean arterial pressure and cerebral perfusion pressure [106]. Phase III trials for cyclosporin A are being planned [10]. As an immunosuppressant drug, cyclosporin A may exhibit adverse effects on the immune system after prolonged use [107]. Other potential limitations include poor brain penetration and a biphasic dose-response curve [107].

#### 5.1.5. Substance P (SP) Antagonists

Substance P (SP) is a neuropeptide released following TBI and contributes to edema and functional deficits [80,108]. Attenuation of TBI-induced SP generation, by preventing its release or antagonizing the neurokinin-1 (NK-1) receptor, reduced inflammation and maintained the integrity of the blood-brain barrier [80]. Administration of the SP (NK-1) antagonist *N*-acetyl-l-tryptophan after experimental TBI reduced vascular permeability and edema formation, and improved motor and cognitive outcomes [108].

#### 5.1.6. Cell Cycle Inhibitors

TBI induces cell cycle activation (CCA) in neurons, and glia; this can result in apoptosis of post-mitotic cells (neurons and mature oligodendroglia), as well as the proliferation and activation of mitotic cells such as astroglia and microglia. In proliferating cells, the cell cycle is controlled by complex molecular mechanisms and progression through distinct phases that require sequential activation of a large group of Ser/Thr kinases called the cyclin-dependent kinases (CDK) and their positive regulators (cyclins) [109]. CCA following TBI may initiate multiple secondary injury mechanisms that contribute to neuronal apoptosis and delayed neurotoxicity. Central or systemic administration of the semi-synthetic flavonoid and non-selective CDK inhibitor flavopiridol reduced lesion size, improved cognitive and sensorimotor outcomes and inhibited caspase-mediated cell death [81,82]. Roscovitine, a more selective inhibitor of CDKs, improved functional recovery, reduced lesion size, attenuated apoptotic pathways, and inhibited progressive neurodegeneration and chronic neuroinflammation in multiple models of TBI [83,84]. More recently, an N6-biaryl-substituted derivative of roscovitine, called CR8, was synthesized [110]. Central as well as systemic administration of CR8, at a dose 10 times less than previously required for roscovitine, significantly improved cognitive outcomes, reduced lesion size and improved neuronal survival after CCI in mice [85]. Several of these CDK inhibitors have been extensively studied as treatment for various neoplasias. Although they are highly toxic when administered chronically, only short-term treatment is necessary for optimal treatment of experimental TBI.

#### 5.1.7. Metabotropic Glutamate Receptor-5 Agonists

Metabotropic glutamate receptor member 5 (mGluR5) is highly expressed in microglia and astrocytes [86,111], as well as in neurons. The mGluR5 selective agonist (RS)-2-chloro-5- hydroxyphenylglycine (CHPG) inhibits caspase-dependent apoptosis across many *in vitro* models. CHPG also strongly attenuates microglial activation, an effect mediated in part through inhibition of reduced nicotinamide adenine dinucleotide phosphate (NADPH) oxidase [86]. Early treatment with CHPG, administered intracerebroventricularly (i.c.v.), shows strong neuroprotection after TBI [87]. Remarkably, CHPG administered one month after CCI in mice significantly reduced expression of reactive microglia expressing NADPH oxidase subunits; decreased hippocampal neuronal loss and lesion progression, as measured by repeated T2-weighted magnetic resonance imaging (at one, two and three months); white matter loss, as measured by high field *ex vivo* diffusion tensor imaging at four months; and significantly improved motor and cognitive recovery [18]. These findings not only highlight the neuroprotective potential of this novel pharmacological treatment for TBI, but also markedly extend the currently-accepted therapeutic window for neuroprotection.

#### 5.1.8. Novel Strategies for Targeting Multiple Cell Death Mechanisms

Programmed neuronal cell death contributes to secondary injury and delayed tissue loss after TBI. Both caspase-dependent and caspase-independent apoptotic mechanisms have been strongly implicated in post-traumatic neuronal cell loss. Caspase-dependent mechanisms are activated in response mitochondrial cytochrome c release into the cytosol where it forms a caspase-activating complex (apoptosome) with Apaf-1 further causing sequential activation of caspase-9 and caspase-3 (the main executioner caspases) [88,112–115]. Caspase-independent mechanisms may be initiated by mitochondrial release of other cell death modulators such as the apoptosis-inducing factor (AIF) [88,116–118]. The AIF-mediated cell death pathway involves its translocation to the nucleus, a step that depends on its interaction with cyclophilin A (CypA), which transports AIF from the cytosol to the nucleus [88,116–118]. Although a direct injection of CypA was reported to reduce blood brain barrier permeability and tissue damage in a stab wound model of brain injury [119], constitutive CypA knockouts were observed to improve long-term functional outcomes, reduce lesion size, improve neuronal recovery and attenuate microglial activation in the CCI model [88]. Furthermore, neurons from *CypA* knockout mice were observed to be protected in *in vitro* models of AIF-mediated cell death and the effects were unrelated to caspase activation [88]. Importantly, inhibition of one type of cell death can enhance other cell death pathways [88]. Both caspase-dependent and AIF-dependent modulation strategies improve outcome after experimental TBI, and combined treatment approaches had additive protective effects [88]. Seventy kilodalton (kDa) HSPs (HSP70s) are stress-induced molecules that are induced in response to CNS and have neuroprotective properties [120,121]. Sabirzhanov *et al*. [89], showed neuroprotective effects of HSP70 overexpression by transfection with HSP70-expression plasmids in multiple *in vitro* models of neuronal cell death. The neurons transfected with HSP70 construct demonstrated significantly reduced expression of markers of caspase-dependent as well as AIF-mediated cell death [89]. Induction of HSP70 using geranylgeranylacetone, before or after TBI in mice, significantly improved outcome (our 2013) [90].

Another cell death mechanism implicated in pathophysiology of TBI is autophagy. Autophagy is a homeostatic and catabolic process that mediates the turnover of bulk cytoplasmic constituents including organelles and protein aggregates in a lysosome-dependent manner, and protects organisms from a variety of diseases, including neurodegeneration. Although autophagy has been shown to be up-regulated after TBI, its function in this context remains controversial [122,123]. Treatment with the anti-oxidant gamma-glutamylcysteinyl ethyl ester (GCEE) after TBI in mice reduced oxidative stress, attenuated autophagy and improved functional outcomes and TBI-induced oxidative stress was observed to be contributing to the overall neuropathology by mediating autophagy [123]. In contrast, rapamycin-induced inhibition of the mammalian target of rapamycin (mTOR) [124] induced autophagy, improving functional recovery and neuronal survival [124]. Therefore, a potential role for modulating autophagy as a neuroprotective strategy requires further study.

#### 5.1.9. Non-Pharmacological Approaches Such As Physical Activity and Exercise

Both pathophysiological changes and neurological impairment after experimental TBI can be attenuated by physical activity [125–127]. The mechanisms underlying the therapeutic effects of exercise may include up-regulation of brain-derived neurotrophic factor (BDNF), leading to enhanced neuronal plasticity as well as anti-apoptotic and anti-inflammatory effects [126,127]; Other factors implicated include cyclic-AMP response-element-binding protein (CREB), protein kinase C (PKC), calcium-calmodulin-dependent protein kinase II (CAMKII), mitogen-activated protein (MAP) kinase I and II (MAPKI and MAPKII) and synapsin-I following [126]. An important variable appears to be the timing of initiation of exercise as a function of injury severity, which can affect the neurotrophic factor response to injury [91,125]. Late initiation of exercise beginning at 5 weeks after CCI in mice, but not early initiation of exercise at 1 week, significantly reduced working and retention memory impairments at 3 months, and decreased lesion volume [91]. The improvement in cognitive recovery was associated with attenuation of classical inflammatory pathways, activation of alternative inflammatory responses and enhancement of neurogenesis [91]. In contrast, early initiation of exercise did not alter behavioral recovery or lesion size, and increased the neurotoxic pro-inflammatory responses [91].

## 6. Conclusions

TBI causes both acute and chronic neurodegeneration by inducing diverse, delayed biochemical changes. Despite strong preclinical evidence supporting neuroprotection treatment to reduce secondary injury, all clinical trials to date have failed. There are many methodological differences between clinical and preclinical studies that may explain this therapeutic discrepancy. More recently, basic science investigators have employed multipotential treatment strategies, rather than more focused modulation, with several of the newer approaches showing robust therapeutic effects across models and species. Moreover, several of the proposed multipotential treatments are already used clinically for other conditions. In addition, delayed exercise intervention looks promising for reducing chronic inflammatory changes and associated neurodegeneration after TBI. Recent advances in clinical trials design, including adaptive design methodology, as well as appreciation for the need for larger sample sizes and more extensive preclinical pharmacological evaluation, may serve to increase the likelihood of successful clinical translation in the future.

## Figures and Tables

**Table 1.** Translational challenges and corrective recommendations.

Translational (preclinical & clinical) challenges	Recommended corrective measures
The diversity and complexity of secondary injury mechanisms	Better elucidating secondary injury mechanisms including diversity of cell death mechanisms and interactions
Inconsistency and inaccuracy of clinical outcome measures and biomarkers	Development of a more comprehensive and symptom-based classification for evaluation of specific behavioral outcomes, quality of life, physiological and imaging-based biomarkers
Variable experimental factors such as multiple injury models of different injury severity, species, strains, genders, ages *etc.*	Evaluation of potential neuroprotective therapies in multiple TBI models, different strains and species—including higher gyrancephalic species, both genders and young *versus* aged animals
Lack of clinically-relevant behavioral outcomes under pre-clinical settings	Use of well-characterized behavioral and histological outcome measures to assess long-term effects of the treatment
Limited preclinical pharmacological evaluation	Examination of pharmacokinetics, pharmacodynamics and brain concentration of the proposed treatment
Inadequate therapeutic window data	Performing therapeutic window studies for prospective neuroprotective treatments to include a more delayed clinically-relevant time points of administration
Inconsistency in statistical modeling/methodologies and inadequate sample sizes	Reducing discrepancies in research methodology between animal and clinical trials, enlargement of sample sizes and use of adaptive design to improve power

**Table 2.** Multipotential novel neuroprotective strategies for TBI.

Emerging neuroprotective approaches	Mechanisms of action/neuroprotective effects
Progesterone	Attenuates glutamate excitotoxicity [51], membrane lipid peroxidation [52], apoptotic and inflammatory pathways [53], and diffuse axonal injury [54].
Thyrotropin-releasing Hormone	Increases cerebral blood flow and metabolism; attenuates peptidyl leukotrienes, platelet-activation factor, endogenous opioids and glutamate [61].
Erythropoietin	Limits excitotoxic, pro-oxidant, edematous, and inflammatory effects [62,63].
Hypothermia	Reduces contusion volume and improves functional outcomes in experimental TBI [64–68], reduces intra-cranial pressure [69–71], and cerebral metabolic rate [72] and increases brain tissue and jugular vein oxygenation [71] in clinical cases, although primary outcome in a major randomized trial not significantly improved [73].
Diketopiperazines	Attenuates cell cycle, calpain, cathepsin; increases BDNF, HSP 70 [74].
SUR1-regulated NC_Ca-ATP_ Channel Inhibitors (glibenclamide)	Reduces edema, secondary hemorrhage, inflammation, apoptosis and lesion size [75].
Statins (rosuvastatin and atorvastatin)	Reduces IL-6, TNF-α, and ICAM-1, glial cell activation and cerebral edema, and restores blood-brain barrier integrity [76,77].
Cyclosporin A	Preservation of mitochondrial function, inhibition of lipid peroxidation and oxidative stress [78,79].
Substance P (SP) Antagonists	Reduced inflammation and maintenance of blood-brain barrier integrity [80].
Cell Cycle Inhibitors	Inhibition of cell cycle activation, neurodegeneration and chronic neuroinflammation microglial and astrocyte activation [81–85].
Metabotropic Glutamate Receptor-5 Agonists (CHPG)	Reduces expression of inducible nitric-oxide synthase, production of nitric oxide and TNF-α, and intracellular generation of reactive oxygen species, limits caspase dependent apoptosis [18,86,87].
Combined inhibition of multiple cell death pathways (e.g., HSP 70)	Limiting both caspase–dependent and caspase-independent cell death [88–90].
Non-pharmacological approaches such as delayed initiation of exercise	Attenuates classical inflammatory pathways, activation of alternative inflammatory responses and enhancement of neurogenesis, increases BDNF [91].
